# A Rare Case of Neonatal Complicated Appendicitis in a Child with Patau's Syndrome

**DOI:** 10.1155/2014/671706

**Published:** 2014-09-03

**Authors:** Valentina Pastore, Fabio Bartoli

**Affiliations:** Pediatric Surgery Unit, Medical and Surgical Sciences Department, University of Foggia, Viale Pinto 1, 71122 Foggia, Italy

## Abstract

Neonatal appendicitis is a rare condition with high mortality rate. Signs and symptoms are often nonspecific, imaging modalities are not always diagnostic, and preoperative diagnosis is difficult with subsequent delay and complications. Its pathophysiology may be different from appendicitis in older children and comorbidities can be found. We report a case of a female neonate with Patau's syndrome, intestinal malrotation, and Fallot tetralogy in whom perforated appendix, probably occurring during fetal period due to vascular insufficiency, was found at laparotomy.

## 1. Introduction

Despite the fact that acute appendicitis is a common diagnosis and is the most common indication for urgent abdominal surgery in children, it is very rare in neonates and shows a high mortality rate, which remains as high as 34% especially in perforated cases [1]. Incidence has been reported to be 0.04–0.2% and male neonates with prematurity or comorbidities (Hirschsprung disease, cystic fibrosis, cardiac defects, tracheoesophageal fistula, and inguinal hernias) are more often affected [2, 3]. Overall, during the last century, about 100 cases have been described [1] and, given its rarity and nonspecific signs and symptoms, preoperative diagnosis is quite difficult with subsequent delay and complications. We report a case of a female neonate with Patau's syndrome, intestinal malrotation, and Fallot tetralogy in whom perforated appendix was found at laparotomy.

## 2. Case Presentation

**Figure 1:**
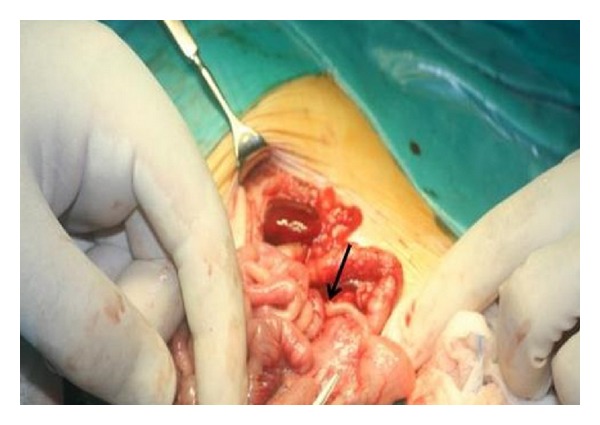
Appendix with perforation near the tip.

**Figure 2:**
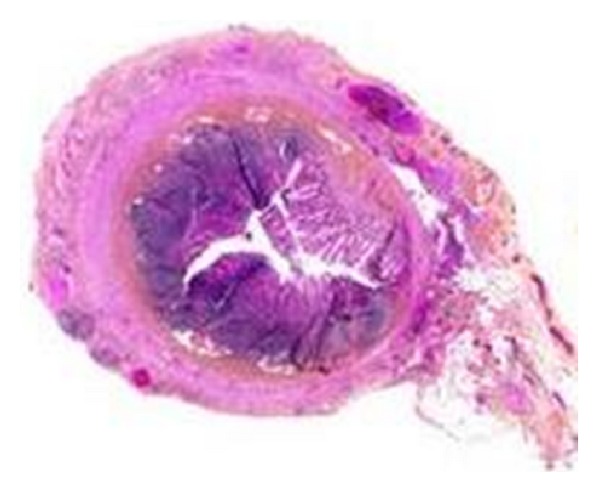
Histological examination of the appendix showing concretions, dense fibrinosuppurative infiltrate, and transmural granulation at the site of perforation.

A female baby, born at 39-week gestation by cesarean section to a second gravid mother with an uncomplicated prenatal history, weighing 2850 gr, was admitted to NICU immediately after birth due to dysmorphia (hands claw and bilateral microphthalmia and aplasia cutis). White blood cell count and CRP were normal, abdomen was palpable, and she suffered only from a slight respiratory distress. In the suspect of a karyotypic abnormality, a cardiac ultrasonography with Doppler was done and a picture compatible with Fallot tetralogy was found. Abdominal US and X-ray showed no evidence of free air and only a slight peritoneal effusion. Chest X-ray was normal. Head MR showed thinning of* corpus callosum*. Three days after admission, general clinical conditions worsened with tachycardia, fever, and abdominal distension. Baby refused food and bile-stained vomiting started. White blood cell count was 17.6 × 10^3^ U/L (neutrophil 77%) and CRP was elevated. The renal function tests were within normal limits. Antibiotic and supporting therapy were started and, in the suspect of bowel intussusception, an abdominal US was done. No ecographic typical signs (apart from a perivesical fluid collection) were found. Then, the baby underwent a barium enema, which showed a bowel malrotation. This was considered the reason of the acute abdomen and a laparotomy was immediately started. Surgical findings included bowel malrotation, dilated small bowel coated with purulent exudates, gangrenous appendicitis with perforation near the tip, and turbid fluid in the peritoneal cavity (Figure 1). Furthermore, there were strong adhesions around the appendix. Ladd's procedure, appendectomy, and toilet of the peritoneal cavity were done. Histological examination of the appendix showed that the lumen was filled with concretions with dense fibrinosuppurative infiltrate and transmural granulation at the site of perforation (Figure 2). Postoperative management was regular: antibiotic and supporting therapy were continued, Patau's syndrome was confirmed as result of karyotype examination, clinical conditions remained stable, and feeding started three days after surgery. White blood cell count switched back to standard rate, CRP normalized, and no changes on cardiac US appeared. Surgical procedure for correction of Fallot syndrome was scheduled but two months after birth the baby suffered from a sepsis due to Klebsiella pneumoniae and she suddenly died.

## 3. Discussion

Neonatal appendicitis is a very rare condition, with no more than 100 cases described over the last century and high mortality and perforation rates [1]. The low incidence can be due to different factors such as the presence of a fetal form of the appendix (funnel shaped with wide opening into the cecum), a liquid diet, recumbent posture, and rare infections [4]. Its pathophysiology may be different from appendicitis in older children and authors think that it could be considered as a localized form of NEC (especially in otherwise healthy neonate) [5], a* morbus sui generis* [6], or that it could be secondary to comorbidities such as Hirschprung disease, cardiac anomalies, tracheoesophageal fistula, cystic fibrosis, or infective diseases (cytomegalovirus and chorioamnionitis) [3]. Since signs and symptoms are nonspecific, a preoperative diagnosis is very difficult and most of neonates have been diagnosed intraoperatively. In fact, all the authors emphasize the difficulty of diagnosis considering both the nonspecific signs and symptoms and the rarity of the disease at this age [2, 7]. Generally, the babies affected may show irritability, distressed breathing, wriggling, swelling of the scrotum, a right lower quadrant palpable mass, abdominal distension, bilious vomiting, erythematous rash over the abdominal wall, and also anorexia, fever, and leukocytosis [3]. As the signs and symptoms are not characteristic, the incidence of perforation is high and is a significant factor in determining the prognosis. Other reasons are to be found in thin appendiceal wall and indistensible cecum. Furthermore, a relatively small undeveloped and functionally nonexistent omentum and small size of the peritoneal cavity allow a more rapid and diffuse contamination and little physiological reserve and are important factors contributing to this high morbidity and mortality rate associated with peritonitis in infants [8]. In the neonate we report, Patau's syndrome was diagnosed based on chromosome testing after a suspected clinical examination. Patau's syndrome, or trisomy 13, affects 1 : 10000–21700 live births and presents abnormalities of nervous system, musculoskeletal, cutaneous, and urogenital and cardiac systems. Evaluation of symptoms and signs was very difficult and in fact, having excluded intussusception (negative abdominal US and barium enema) and believing she could suffer from an acute abdomen due to bowel malrotation, laparotomy was done about 12 hours after diagnostic examinations. We found bowel malrotation but the acute abdomen was due to the perforated appendix with secondary peritonitis. Retrospectively examining the signs we had found before surgery, we think that, even if the clinical conditions worsened three days after birth, peritoneal effusion shown on abdominal US made few hours after birth could have been considered an initial sign. Furthermore, at laparotomy, there were strong adhesions around the appendix and these can justify a pre- or perinatal perforation and negative preoperative abdominal US and barium enema. About the possible aetiologies of appendiceal perforation in our patient, the most probable could be the vascular insufficiency (considered a major cause of perforation) which might have induced poor circulation before the onset of symptoms [3, 9]. Surgical approach was the standard Ladd's procedure, classical appendectomy, and peritoneal toilet without intraoperative complications. Postoperative course was uneventful strictly considering the surgical management and the baby started feeding three days after surgery. Postoperative laboratory findings were normal and clinical conditions remained stable. However, Patau's syndrome carries a high mortality rate with more than 89% of the children dying before hospital discharge and comfort care is the treatment of choice for most of them [10]. In fact, about two months after surgery, our baby became septic, not responsive to antibiotic therapy, and she suddenly died. In conclusion, acute appendicitis is a rare condition in neonates and is rarely diagnosed preoperatively because symptoms are not specific. Delay in diagnosis carries a high perforation and complication rate, also because diagnostic examinations are not always comprehensive, even if we believe that in our patient perforation occurred during fetal period. In order to reduce mortality, in doubtful cases, appendicitis should also be considered in the differential diagnosis especially in neonates with comorbidities.
